# Non-uniform dendritic distributions of *I*_h_ channels in experimentally-derived multi-compartment models of oriens-lacunosum/moleculare hippocampal interneurons

**DOI:** 10.1186/1471-2202-15-S1-P43

**Published:** 2014-07-21

**Authors:** Vladislav Sekulić, Tse-Chiang Chen, John J Lawrence, Frances K Skinner

**Affiliations:** 1 Toronto Western Research Institute, University Health Network, Toronto, Ontario, M5T 2S8, Canada; 2 Department of Physiology, University of Toronto, Ontario, M5S 2J7, Canada; 3 Department of Medicine (Neurology), University of Toronto, Ontario, M5S 2J7, Canada; 4 Department of Medical Biophysics, University of Toronto, Ontario, M5S 2J7, Canada; 5 Department of Biomedical and Pharmaceutical Sciences, University of Montana, Missoula, Montana, 59812, USA; 6 NIH COBRE Center for Structural and Functional Neuroscience, University of Montana, Missoula, Montana, 59812, USA

## 

Inhibitory interneurons are crucial for generating prominent network rhythms and coordinating information flow in hippocampal microcircuits. The oriens-lacunosum/moleculare (O-LM) cell is an interneuron type in the hippocampal CA1 region that synapses onto distal dendrites of pyramidal cells [1]. O-LM cells mediate feedback inhibition onto pyramidal cells and gate information flow between sensory input from entorhinal cortex and previously stored associations from the CA3 area. Despite the distal location of inhibitory synapses from O-LM cells onto the excitatory populations, their control of pyramidal cell output has been clearly shown [2]. Thus, how the dynamic firing properties of O-LM cells in its network circuit environment is generated needs to be understood. Furthermore, it is clear that the presence and distribution of voltage-gated channels on the dendrites of O-LM cells would affect its integrative properties in response to synaptic input. However, given the highly challenging aspects to experimentally determine whether and what sort of distributions of voltage-gated channels are present on dendrites, we take advantage of computational modeling studies to consider different possibilities.

In this work, we focus on *I*_h_ channels. While the existence of *I*_h_ channels in O-LM cells has long been known [3], it is at present unknown whether these channels are present on O-LM cell dendrites. In previous work, we used ensemble modeling techniques in conjunction with experimental data to show that physiologically realistic multi-compartment O-LM cell models may possess dendritic *I*_h_, but only uniform distributions across the dendritic tree were examined. In the work here, we turned our focus to how the kinetics of *I*_h_ and non-uniform distributions would affect our models’ output. In tuning our models, we found that different *I*_h_ kinetics as well as non-uniform distributions were better able to reproduce experimental O-LM cell responses. Interestingly, this occurred only when there were decreasing conductance densities away from the soma. This is in contrast to pyramidal cells which have higher *I*_h_ conductance densities in more distal dendrites [4]. Non-uniform distributions of *I*_h_ would indicate that there are particular synaptic input distributions that affect the firing properties of O-LM cells and thus their ability to affect information flow.

## References

[B1] Freund T Buzsáki G Interneurons of the hippocampus Hippocampus 1996 15 347 470 8915675 10.1002/(SICI)1098-1063(1996)6:4<347::AID-HIPO1>3.0.CO;2-I

[B2] Leão RNR Mikulovic SS Leão KEK Munguba HH Gezelius HH Enjin AA Patra KK Eriksson AA Loew LML Tort ABLA OLM interneurons differentially modulate CA3 and entorhinal inputs to hippocampal CA1 neurons Nat Neurosci 2012 15 1524 1530 10.1038/nn.3235 23042082 PMC3483451

[B3] Maccaferri G McBain CJ The hyperpolarization-activated current (Ih) and its contribution to pacemaker activity in rat CA1 hippocampal stratum oriens-alveus interneurones J Physiol 1996 15 119 130 8951716 10.1113/jphysiol.1996.sp021754 PMC1160917

[B4] Magee JC Dendritic Hyperpolarization-Activated Currents Modify the Integrative Properties of Hippocampal CA1 Pyramidal Neurons J Neurosci 1998 15 7613 7624 9742133 10.1523/JNEUROSCI.18-19-07613.1998 PMC6793032

